# Pediatric Trauma Transfer Imaging Inefficiencies—Opportunities for Improvement with Cloud Technology

**DOI:** 10.3934/publichealth.2016.1.49

**Published:** 2016-02-26

**Authors:** Yana Puckett, Alvin To

**Affiliations:** 1 Saint Louis University College of Public Health and Social Justice, Saint Louis, MO, USA; 2 Saint Louis University School of Medicine, Saint Louis, MO, USA

## Abstract

**BACKGROUND:**

This study examines the inefficiencies of radiologic imaging transfers from one hospital to the other during pediatric trauma transfers in an era of cloud based information sharing.

**METHODS:**

Retrospective review of all patients transferred to a pediatric trauma center from 2008–2014 was performed. Imaging was reviewed for whether imaging accompanied the patient, whether imaging was able to be uploaded onto computer for records, whether imaging had to be repeated, and whether imaging obtained at outside hospitals (OSH) was done per universal pediatric trauma guidelines.

**RESULTS:**

Of the 1761 patients retrospectively reviewed, 559 met our inclusion criteria. Imaging was sent with the patient 87.7% of the time. Imaging was unable to be uploaded 31.9% of the time. CT imaging had to be repeated 1.8% of the time. CT scan was not done per universal pediatric trauma guidelines 1.2% of the time.

**CONCLUSION:**

Our study demonstrated that current imaging transfer is inefficient, leads to excess ionizing radiation, and increased healthcare costs. Universal implementation of cloud based radiology has the potential to eliminate excess ionizing radiation to children, improve patient care, and save cost to healthcare system.

## 1. Introduction

Pediatric trauma patients are often transferred from rural outside hospitals (OSH) to accredited trauma centers for definitive treatment. This transfer is often unnecessary delayed for additional diagnostic evaluation, including radiographic studies [1]–[6]. Imaging prior to transfer delays the care of the patient by increasing the amount of time it takes to upload imaging. Imaging is typically sent with the patient on a CD-ROM, an outdated form of information transfer invented in the 1980s. Oftentimes, imaging is unable to be uploaded or the image is lost en-route resulting in repeat imaging of child. This exposes children to excess ionizing radiation.

Ionizing radiation in children has been proven to be a risk factor for malignancy in the future. Evidence from studies conducted following the Chernobyl accident, nuclear tests, environmental radiation pollution and indoor accidental contamination reveals consistently increased chromosome aberration and micronuclei frequency in exposed than in referent children [10]–[14].

In the age of cloud-based computing, which enables the use of virtually unlimited online server storage space, imaging could be viewed instantly and amount of radiation can be reduced dramatically [7]–[9]. In addition, radiation exposure can be tracked for every individual in a centralized imaging system. However, making imaging available for use in cloud has not been prevalent throughout the United States and outdated form of image transfer continues to persist.

Our study aims to evaluate how often imaging for pediatric trauma patients transferred to our designated trauma center was performed at the outside hospital, how often imaging accompanied the patient, how often imaging was unable to be uploaded to the system, how often imaging had to be repeated, and how long the trauma transfer took place.

## 2. Methods

We conducted a retrospective chart analysis of all patients transferred from outside medical facilities to our Level I Pediatric Trauma Care center from 2008 to 2014. A total of 1761 charts met inclusion criteria. Inclusion criteria included age between 0–21, all race, all gender.

We evaluated the charts for demographic information of the transferred patient; whether imaging was performed at the outside facility; whether imaging was sent with the patient; whether imaging could have uploaded onto the computer system at the accepting facility; whether repeat imaging had to be performed, whether computed tomography (CT) scans were performed unnecessarily, whether laboratory results taken at outside facility were sent with the patient or had to be repeated. Statistical Package for the Social Sciences (SPSS) Version 21 (SPSS, Chicago, IL) was used for statistical analysis.

## 3. Results

A total of 1761 patients met inclusion criteria and were transferred to our Level I Pediatric Trauma Care center from 103 unique OSHs. The mean age of trauma transfer was 6.8 years (SD 5.1). Males comprised 64% (1132/1761) of the transfers. Falls resulting in fractures were the most common cause of trauma (77% 1358/1761). The average time it took from time of arrival to the outside facility to departure was 155.35 minutes (SD 90.3).

Imaging was performed at the outside hospital 32% (559/1761) of the time. Imaging was sent with the patient 91.4% (511/559) of the time. Imaging was unable to be uploaded to electronic records at the accepting facility 31.9% (163/511) of the time. For patients that were transferred with imaging data, labs had to be repeated 6.7% (34/511) of the time. X-rays and CTs were repeated 18.9% (97/511) and 1.8% (9/511) of the time at the accepting facility. 63% (61/97) and 67% (6/9) of these scans respectively were repeated when it was not possible to upload OSH results from the provided CD-ROM.

## 4. Discussion

Cloud based radiology systems have become widely available and have been implemented in several small private hospitals throughout the United States and several studies show that cloud technology can be extremely cost effective if implemented nationwide [15]–[17]. The majority of hospitals do not use cloud based radiology and rely on archaic methods of imaging transfer such as CD-ROMs. The reason for this is unclear but has been speculated to be due to issues with the Health Insurance Portability and Accountability Act of 1996 (HIPAA) compliance and the financial investments of hospitals in the currently utilized radiology systems [15]–[17].

Our Level I Pediatric Trauma Care center receives patients transferred from 103 OSHs. Imaging results conducted at the initial OSH are concurrently delivered on CD-ROMs as patients are admitted. This method of inter-facility data transfer is direct; however, it introduces vulnerabilities, such as risks of data loss, data corruption, or incompatibility. Our findings demonstrate that in 31.9% of cases when imaging was transferred by CD-ROM, it was not possible to upload the data to our system. Patients transferred to our facility required subsequent screening by x-ray (18.9%) and CT (1.8%). Importantly, 63% and 67% of repeated imaging respectively were conducted on patients who had previous results that were inaccessible by CD-ROM upload. This imperfect medium for data transfer causes delays in time-to-treatment and may worsen patient prognosis. These repeat screens not only burden the healthcare system, but moreover, re-expose patients unnecessarily to ionizing radiation. The genetic damage and increased predisposition for future malignancies from multiple exposures to radiation has been significantly documented and should be minimized when possible [10]–[14].

Maintaining electronic medical records (EMR) on a centralized cloud storage system may facilitate fast and reliable inter-facility transfer and reduce the frequency of avoidable repeated screens. Furthermore, a central repository may provide additional data transfer advantages that are unachievable with the current practice of local site storage and transfer via CD-ROMs. Centralized data stored on the cloud can be updated in real-time as results are collated and viewed concurrently by multiple healthcare providers. This may benefit patients—especially those in critical condition—as they are transferred between facilities by allowing awaiting healthcare teams to view screening results before patients arrive and preemptively strategize actions. EMRs stored simultaneously on multiple remote servers can also increase fidelity and is less prone to data loss compared with the vulnerability of transporting a single physical CD-ROM.

The national movement towards EMRs began when the Patient Protection and Affordable Health Act in 2010 was enacted. The transition from paper charting to EMRs was, however, filled with doubt and similar worries about personal health information security [18]–[21]. Storage of electronic records using cloud solutions has had even greater resistance (22). Security and privacy concerns need to be adequately addressed with sufficient measures including data encryption, digital signatures, access monitoring, and compliance with security requirements (23). Despite the gradual adoption, five decades of data strongly support that paper based charting is inefficient and outdated [18]–[24]. Since March 2014, all hospitals have implemented EMRs and preliminary studies demonstrate improvement in the care of patients [25].

Our study demonstrates measurable inefficacies of redundant screenings that occur with current data transfer practices used during inter-facility trauma patient transfer. Healthcare administrators and policy makers should take into account the negative effects of redundant screenings to patient outcomes and the healthcare system when weighing the benefits of a universal cloud based system against the risks to data security and privacy. A secured and centralized cloud based storage of imaging results may augment authorized access, reduce the incidence of redundant screenings, streamline patient healthcare, and improve the outcomes of all patients.

Some limitations of this study need to be recognized. The specific nature of unsuccessful CD-ROM uploads was not subcategorized in the available data. Furthermore, the rational for repeat imaging screens were not reported systematically and therefore not analyzed in this study. Repeat tests may have been conducted for reasons other than failed data upload, such as monitoring trauma progression or examining ranges outside of the scope of prior scans. The observed overabundance of repeat scans in cases with failed data transfer may be caused by other factors that were unaccounted for.
